# Evolutionary fitness as a function of pubertal age in 22 subsistence-based traditional societies

**DOI:** 10.1186/1687-9856-2011-2

**Published:** 2011-06-21

**Authors:** Ze'ev Hochberg, Aneta Gawlik, Robert S Walker

**Affiliations:** 1 Division of Pediatric Endocrinology, Meyer Children's Hospital, Rambam Medical Center, and Rappaport Faculty of Medicine and Research Institute, Technion-Israel Institute of Technology, Haifa, Israel; 2 Department of Pediatric Endocrinology and Diabetes, Medical University of Silesia, Katowice, Poland; 3 Department of Anthropology, University of Missouri, Columbia MO 65211, USA

## Abstract

**Context:**

The age of puberty has fallen over the past 130 years in industrialized, western countries, and this fall is widely referred to as the secular trend for earlier puberty. The current study was undertaken to test two evolutionary theories: (a) the reproductive system maximizes the number of offspring in response to positive environmental cues in terms of energy balance, and (b) early puberty is a trade-off response for high mortality rate and reduced resource availability.

**Methods:**

Using a sample of 22 natural-fertility societies of mostly tropical foragers, horticulturalists, and pastoralists from Africa, South America, Australia, and Southeastern Asia, this study compares indices of adolescence growth and menarche with those of fertility fitness in these non-industrial, traditional societies.

**Results:**

The average age at menarche correlated with the first reproduction, but did not correlate with the total fertility rate TFR or reproductive fitness. The age at menarche correlated negatively with their average adult body mass, and the average adult body weight positively correlated with reproductive fitness. Survivorship did not correlate with the age at menarche or age indices of the adolescent growth spurt. The population density correlated positively with the age at first reproduction, but not with menarche age, TFR, or reproductive fitness.

**Conclusions:**

Based on our analyses, we reject the working hypotheses that reproductive fitness is enhanced in societies with early puberty or that early menarche is an adaptive response to greater mortality risk. Whereas body mass is a measure of resources is tightly associated with fitness, the age of menarche is not.

## Introduction

The age of puberty has fallen over the past 130 years in industrialized, western countries, where menarche age has receded from 16.5 years in 1880 to the current 12.5 years in western societies; this decline has occurred concomitantly with an improvement in child health [1]. The progressively declining age of thelarche and menarche may have multiple explanations. Primates' studies suggest a role for prenatal androgens and social factors (like the social rank) [2,3]. In the last decade, a popular notion among investigators is that early puberty may result from environmental exposure to endocrine disrupting chemicals (EDC), thus accelerating hypothalamic maturation [4]. Whereas EDC may have a bearing on the earlier age of thelarche, evolutionary forces may add a new angel to explain the secular trend in the age of menarche over the last 130 years. Even though the age at menarche is strongly linked to genetic variations [5], the time course of the secular trend suggests strong environmental influence.

This secular trend for early onset of puberty is a topic of much research interest. This trend was recently examined from a life-history evolutionary perspective, and we previously suggested that transition from juvenility to adolescence is a period of adaptive plasticity [6,7]. Two models have been proposed to explain this adaptation. Gluckman and Hanson suggested that life-history strategies for greater reproductive fitness (a product of the fertility rate and survivorship) could account for the current early onset of puberty [8]. The declining age of pubertal development have both been proposed as an adaptive response to positive environmental cues in terms of energy balance [7]. The assumption is that the reproductive system maximizes the number of offspring by balancing the benefit of more births against the costs of maternal mortality. With respect to puberty, women face a trade-off between spending a long time accumulating resources through childhood growth and weight gain in order to improve the likelihood for successful pregnancy, against an early-age reproduction in order to increase the number of reproductive cycles. Indeed, heavy women in pre-industrial societies are more fertile, and both increased body weight and fertility are correlated with high birth rates [9]. This trade-off model has been used to predict that 18 years is the optimal age for first birth, which is near the observed average 17.5 years in such societies [10].

A different evolutionary trade-off was suggested by Migliano *et al *[11]. Based on analysis of the stature, growth, and reproductive fitness for the Aeta and the Batak pygmy from the Philippines, they argued that that the small body size of pygmy populations is an adaptation that evolved as the result of a life history tradeoff between the fertility benefits of a large body size against the costs of late growth cessation, under the circumstances of significant young and adult mortality. They showed that the small pygmy body size evolved through the early onset age of juvenility and adolescence [12], and suggested early cessation of growth is a trade-off for high mortality rate and reduced resource availability. Thus, short life expectancy may be initially a determinant of early puberty but subsequently, early puberty as an adaptive response may determine longer lifespan due to reduced adult size and resource need.

The present study was designed to explore these two hypotheses. To this end, it takes an inter-population approach that compares indices of adolescence (menarche, and the growth spurt) with those of fertility fitness in non-industrial, traditional societies. Using a sample of 22 natural-fertility societies of mostly tropical foragers, horticulturalists, and pastoralists from Africa, South America, Australia, and Southeastern Asia, we hypothesized that (a) early adolescence would be associated with greater reproductive fitness or (b) with mortality risk, and predicted that the age at menarche and indices of pubertal growth will (a) negatively correlate with fertility rate or (b) positively correlate with survivorship. Although the reproductive and life history strategies of males and females are quite distinct in these societies, similar considerations might apply to the males, for which we included indices of adolescent growth spurt in our study.

## Methods

### Natural-fertility human societies

The sample used here is 22 subsistence-based societies and the demographic characteristics of each society, collected at different times during 1967-1988; these have been previously described [13] (Table 1). Information on the life history and population density of these societies and their average height, body weight, and BMI is available at http://anthropology.missouri.edu/people/walker.html as compiled by one of us (RSW) from previous reports [14-16] and sources therein. In these societies, the age of menarche ranged from 12.6-18.4, and the age at growth spurt takeoff ranged from 10-13.5 years.

**Table 1:** Ecology and economy type of the twenty tribes comprising the study populations

Name	Country	Ecology	Economy	Subjects
**Aeta**	Philippines	tropical forest	mixed	365

**Baka **(West Pygmy)	Cameroon	tropical forest	forager	217

**Batak**	Philippines	tropical forest	mixed	36

**Arnhem land**	Australia	coastal/desert	farming-foraging	> 700

**Guaja**	Brazil	neotropical forest	forager	103

**Hadza**	Tanzania	savanna/woodland	forager	> 700

**Hiwi**	Venezuela	savanna/gallery	forager	59

**Ju'/hoansi**	Botswana/Namibia	desert/savanna	forager	278

**Maku-Nadeb**	Brazil	neotropical forest	farming-foraging	97

**Tsimane**	Bolivia	neotropical forest	farming-foraging	603

**Yanomamo**	Venezuela	neotropical forest	farming-foraging	116

**Ache**	Paraguay	neotropical forest	farming-foraging	484

**Casiguran Agta**	Philippines	tropical forest	forager	155

**Aka**	Congo/C.A.R.	tropical forest	forager	186

**Efe **(East Pygmy)	Congo (Ituri)	tropical forest	forager	145

**Gainj & Asai**	New Guinea	highlanders	farming-foraging	153

**Turkana**	Kenya	savanna	pastoral/mixed	417

**Warao**	Venezuela	tropical forest	forager	116

**Gambia**	Gambia	savanna/forest gallery	farming-foraging	> 700

**Toba**	Argentina	savanna/dry forest	mixed	411

**Wichi**	Argentina	savanna/dry forest	mixed	328

**Maya**	Mexico	forest/savanna	mixed	182

The stages of puberty were determined in girls of each society by the mean age of menarche, by the adolescent takeoff height velocity, peak height velocity, and for the end of puberty - the return to weight and height takeoff velocities. Indices of fertility in each society were determined from the average age at first reproduction, the interbirth interval (IBI, by self report), and total fertility rate (TFR). Reproductive fitness was calculated per society from the population average survivorship to age 15 (L15, range 0.33-0.80) and the TFR (range 2.6-9.0), TFR*L15, as previously suggested [17]. Mortality risk was determined from the survivorship to age 15 and life expectancy at birth (range 24.3-47.5) and age 15 (range 29.6-47.0).

At the time of data collection, ethical approval was considered not required, and the authors had no access to individuals' data.

### Statistical analyses

Linear regression analyses with 95% confidence intervals of outcome as a function of IBI were performed using *Statistica 6.0*. For these analyses, the average of each study parameter for each society was a single point in the regression analysis. One-way analysis of variance (ANOVA) was used to determine whether statistical differences exist between the study parameters in the 22 studied societies. Statistical significance was set at p < 0.05.

## Results

### Adolescent age and reproductive fitness

**Figure 1:**
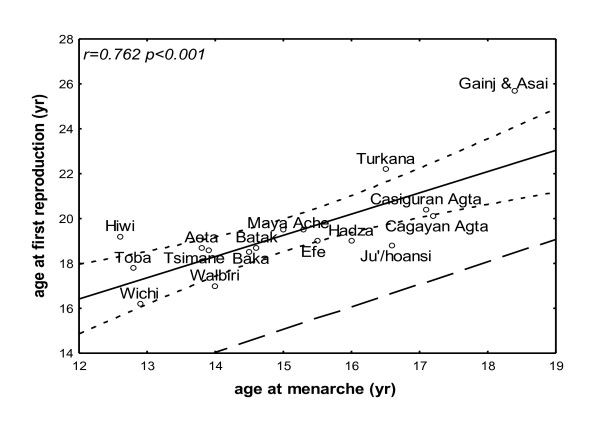
**Age of menarche and the first reproduction**. A regression line and 95% confidence limits for the age of the first reproduction as a function of the menarche age in traditional societies. The dashed line connects identical ages on the X and Y axes, demonstrating the constant age gap between the menarche age and first reproduction age.

**Figure 2:**
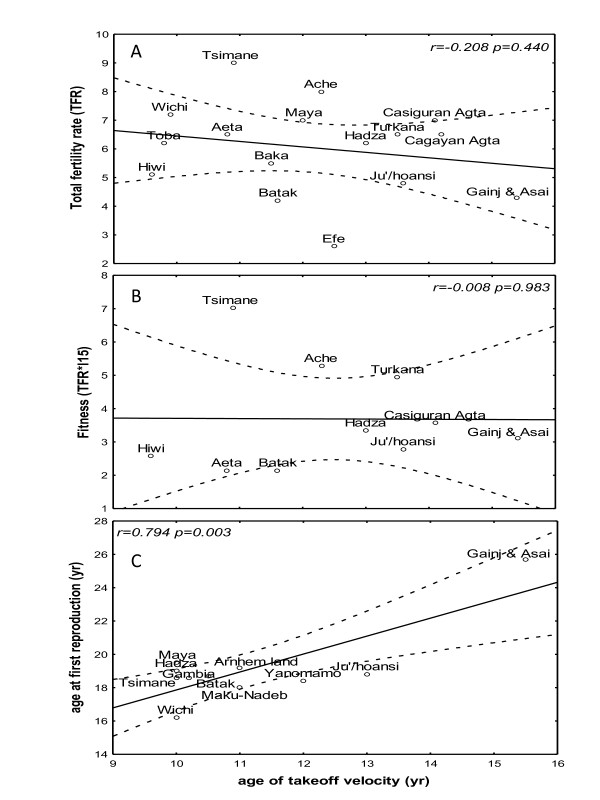
**Puberty and reproductive fitness**. A regression and 95% confidence limits for the age at first reproduction as a function of the height spurt takeoff in girls of traditional societies.

The age at menarche correlated positively with the age at first reproduction (r = 0.762, p < 0.001), and the average duration between the two events was four years (Figure 1). When we tested the hypothesis that early adolescence is associated with increased reproductive fitness, the age at menarche and the growth indices (age at takeoff, peak velocity, and the return to weight and height takeoff velocity) did not correlate with the IBI (Table 2). The age at menarche did not correlate with TFR or reproductive fitness (NS: r = -0.208, p = 0.440; r = -0.008, p = 0.983, respectively), whereas the age at first reproduction correlated positively with the age at height spurt takeoff (r = 0.794, p = 0.003; Figure 2), peak height velocity (r = 0.791, p = 0.011), and return to takeoff height velocity (r = 0.765, p = 0.027), but TFR and reproductive fitness did not correlate with any of these variables (Table 2).

**Table 2:** Insignificant correlations

	Correlations	r	p
***Fitness***	Age at menarche vs IBI	0.248	0.354
	Age at takeoff velocity vs IBI	0.501	0.117
	Age at menarche vs TFR	-0.208	0.440
	Age at menarche vs reproductive fitness	-0.008	0.983
	TFR vs age at takeoff velocity	-0.565	0.089
	Reproductive fitness vs age at takeoff velocity	-0.356	0.489
	Reproductive fitness vs age at peak velocity	-0.457	0.543

***Body size***	Age at menarche vs average adult height	-0.109	0.687
	Average adult height vs age at first reproduction	-0.042	0.856
	Average adult height vs TFR	0.355	0.148
	Average adult height vs reproductive fitness	0.480	0.135
	BMI vs TFR	0.296	0.232
	BMI vs reproductive fitness	0.347	0.295

***Density***	Population density vs age at menarche	0.235	0.486
	Population density vs TFR	0.202	0.530
	Population density vs reproductive fitness	0.141	0.698

***Survivorship***	Survivorship to age 15 vs age at menarche	0.207	0.541
	Life expectancy at birth vs age at menarche	0.146	0.688
	Life expectancy at age 15 vs age at menarche	0.139	0.701

BMI - body mass index, IBI - interbirth interval, TFR - total fertility rate,

### Body size

**Figure 3:**
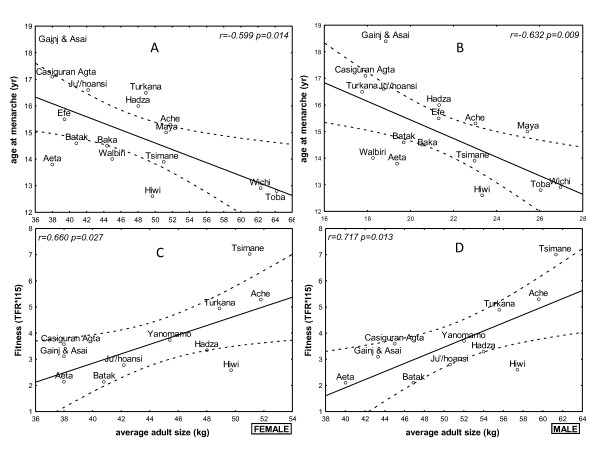
**Puberty and body weight**. *Upper panels*: A regression and 95% confidence limits for the age at menarche as a function of the average adult body weight (*left panel*) and BMI (*right panel*). *Lower panels*: A regression and 95% confidence limits for reproductive fitness as a function of the body weight in both females (*left panel*) and males (*right panel*).

The average age at menarche correlated negatively with the average adult body weight (kg) (r = -0.599, p = 0.014; Figure 3) and body mass index (BMI) (r = -0.632, p = 0.009; Figure 3), but not with the average adult height (Table 2). The average adult body weight (kg) correlated negatively with the age at first reproduction (r = -0.450, p = 0.041), and the average adult body weight (kg) positively correlated with the respective society's reproductive fitness (r = 0.660, p = 0.027; r = 0.717, p = 0.013, respectively; Figure 3). The average adult heights did not correlate with the age at first reproduction, TFR, or reproductive fitness (Table 2). The BMI correlated with the age at first reproduction (r = -0.466, p = 0.033), but not with the TFR or reproductive fitness.

### Adolescent age and the physical environment

The average age at menarche and parameters of the growth spurt for each society did not correlate with their respective population density. The population density correlated positively with the women's age at first reproduction (r = 0.680, p < 0.005), but not with menarche age, TFR, or reproductive fitness (Table 2).

### Adolescent age and survivorship expectancy

When we tested the hypothesis that early adolescence and small body size is a trade-off for high mortality risk, we found that the survivorship to age 15 and life expectancy at birth or age 15 did not correlate with the age at menarche or age indices of the adolescent growth spurt (Table 2). Adding survivorship to a multivariate analysis for age at menarche or age at first reproduction as a function of BMI and population density added no statistical significance.

### Ecological and economic correlates

When the societies were categorizing societies by their ecology, dwellers of the tropical forest, the neotropical forest, and the savanna had comparable ages at menarche and reproductive fitness. Of the studied societies, the TFR was highest among dwellers of neotropical forests (8.3 ± 0.6), as compared to those in the savanna (6.5 ± 0.7) and the tropical forests (5.4 ± 1.7, p = 0.015; Table 3). When the societies were categorized by their economy type, the age at menarche, TFR, and reproductive fitness were not significantly different between the farmers-foragers, the foragers, and the mixed-economy groups (results not shown).

**Table 3:** Ecology type of the societies comprising the study populations

VARIABLES	Tropical forest	Neotropical forest	Savanna/other	p Value
**Age at menarche (n)**	(n = 6) 15.5 ± 1.4	(n = 2) 14.6 ± 1.0	(n = 6) 14.3 ± 1.8	0.45

**TFR (n)**	(n = 6) 5.4 ± 1.7	(n = 3) 8.3 ± 0.6	(n = 7) 6.5 ± 0.7	**0.015**

**Reproductive fitness (n)**	(n = 3) 2.6 ± 0.9	(n = 3) 5.3 ± 1.7	(n = 4) 3.9 ± 1.2	0.09

TFR - total fertility rate, mean ± SD

## Discussion

In considering human adolescence in the context of evolutionary fitness, we considered key traits that were available from data collected in 1967-1988. These include the age of menarche, the pattern and timing of the adolescent growth spurt, the body size, and the determinants of reproductive fitness, namely the age of the first reproduction, the IBI, the number of progeny, and mortality risk [18]. The database contains no information on birth size, which may influence the age at menarche. Extended growth and large body size in humans prompt fertility gains and reduced offspring mortality [19]. Consequently there is a pressure for delayed reproductive onset, whereas early reproduction minimizes the likelihood of death before reproduction.

Several limitations of this approach require consideration. i. By nature of this approach, comparing population level averages and attributing differences to ecological differences may not be straightforward. It assumes that the relationships observed would be the same if examined at an individual level [20]. In their report, Greenland and Robin construct several epidemiological examples that show that data at the ecologic level can be misinterpreted if nonlinear effects at the individual level, and other confounds, are not accounted for. Such data for the individual level were not available to us. ii. Comparative studies across societies may also suffer from problems of phylogenetic non-independence. This was addressed for these data previously by adjusting for geographical location (Africa, South America, Australia and Southeast Asia), but the effect was very weak and not significant in any of the multiple regressions [13].

Pubertal timing is not a univocal parameter; secular changes may occur differently for some pubertal events. Whereas the age of thelarche has reduced in the last decades, changes in menarche have been more subtle and mental maturation - deferred. Also, it is not clear that menarche is a good proxy for fecundity. The age at first reproduction in the present study strongly correlated with menarche, but the potential role of anovulatory menstrual cycles after menarche and potential non-linearity of this trend remain options. For example, it is possible that girls who differ on age at menarche may be similar with regard to age of fecundity; earlier menarche may relate to a greater number of anovulatory cycles [21], and indeed, regular (ovulatory) cycling was shown to follow a secular trend towards delay as opposed to early menarche [22].

The data show that at natural fertility, early adolescence in girls, as assessed by the age of menarche and the growth pattern, corresponds to a young age with the first reproduction occurring about four years later, as previously suggested [23]. However, based on our analyses, we reject the two working hypotheses: reproductive fitness is enhanced in societies with early puberty [8] and early menarche is an adaptive response to greater mortality risk [11].

We focus here on subsistence-based societies because most resources are invested as somatic capital in terms of growth, body size and fertility (reproductive fitness), as opposed to stored and inherited wealth [15]. The relative contribution of biological and behavioral factors in determining natural fertility change with the environment. Environmental factors to consider in an inter-population study include the physical environment (e.g., population density), the biological environment (e.g., food availability, disease, and other mortality risks) and social behaviors (e.g., age at marriage) [15]. We defined reproductive fitness as a function of TFR and L15, as previously suggested [17]; these two variables were selected among other because information on these two parameters was available for almost all of the societies in the database. The age at menarche did not correlate with TFR or reproductive fitness. Whereas reproduction starts early in societies in which puberty occurs early, in the context of high population density [19], their reproductive fitness does not increase. The dwellers of the neotropical forests have a high TFR, but given their mortality risk, they have comparable reproductive fitness to the other ecology groups.

We confirm a previous assertion for greater reproductive fitness among heavier, better-nourished traditional societies [9,24]. When considered as a whole, we found that the average adult body weight, but not height, correlated negatively with age at menarche and the age at first reproduction, and positively with reproductive fitness. The BMI may not work as well in the extremes of size; in very small or very tall populations the BMI is not as accurate as it is in average size populations. Even though, these findings provide indirect support to the hypothesis that early puberty among girls who live in affluent and developed countries is a response to a positive energy balance. Indeed, among contemporary girls in developing countries, the age at menarche among the prosperous is earlier than that of the underpriviledged [1].

Based on the high mortality rates of the Philippine pygmy, Migliano et al suggested that early fertility is part of the "fast" extreme of life history strategies to which the pygmy adapt [25], with both longevity and resource availability as limiting factors [11]. Indeed, early life stress is associated with premature juvenility and adolescence [1,12,26]. The results of the present study do not confirm the fast life history theory; we found no correlation between adult height and the age of menarche with survivorship. Yet, population density correlated with the age of first reproduction, in addition to our previous assertion that body size in a traditional society was dependent upon the population density [19]. We have previously suggested that population density acts through two pathways - nutritional constraints and juvenile mortality - at varying intensities, and can contribute to a nearly twofold range in body size across human societies [19]. The sample of the present study includes two African pygmy groups - the Baka (West pygmy) and the Efe (East pygmy), both of whom are not consistent with the Migliano risk/early fertility model. The average age of menarche and age at first reproduction in these two societies, 14.5 and 15.5, respectively 18.5 and 19, respectively, were close to those of all of the other studied societies [13].

The secular trend for an early age of menarche has been rapid over the past 130 years in developed countries [27]. This trend is rightly interpreted as a reflection of improved nutrition and health in childhood [18]. Life history theory postulates tradeoffs of current versus future reproduction and fertility versus mortality risk. Life-history modeling predicted that a reduction in juvenile mortality reduces the age of menarche [8] or that low survivorship accelerates the life history [11]. Given the close interaction between resource availability and reproduction, we anticipated that those environmental factors that determine late metabolic homeostasis, attainment of adult size and cessation of growth would interface with those influencing the timing of sexual maturation. The data do not support these predictions. Whereas body mass as a measure of resources is tightly associated with fitness in these traditional societies, the age of menarche is not. Thus, it may be that women's physiology tracks its own condition in such a way as to maximize their individual fitness.

## Abbreviations

BMI: Body mass index; IBI: Interbirth interval; EDC: endocrine disrupting chemicals; L15: survivorship to age 15; TFR: Total fertility rate

## Competing interests

The authors declare that they have no competing interests.

## Authors' contributions

ZH contributed to the concept and design, analysis and interpretation of data, writing the article. AC contributed to the design, analysis and interpretation of data. RSW contributed to the concept and design, acquisition and interpretation of data. All authors read and approved the final manuscript.
